# Changes in Body Composition, Energy Metabolites and Electrolytes During Winter Survival Training in Male Soldiers

**DOI:** 10.3389/fphys.2022.797268

**Published:** 2022-02-16

**Authors:** Tarja Nykänen, Tommi Ojanen, Risto Heikkinen, Mikael Fogelholm, Heikki Kyröläinen

**Affiliations:** ^1^Army Academy, Finnish Defence Forces, Lappeenranta, Finland; ^2^Finnish Defence Research Agency, Finnish Defence Forces, Tuusula, Finland; ^3^Statistical Analysis Services, Analyysitoimisto Statisti Oy, Jyväskylä, Finland; ^4^Department of Food and Nutrition, University of Helsinki, Helsinki, Finland; ^5^Faculty of Sport and Health Sciences, University of Jyväskylä, Jyväskylä, Finland; ^6^Finnish Defence Forces, National Defence University, Helsinki, Finland

**Keywords:** military training, energy deficit, fat mass, biomarkers, recovery

## Abstract

The aim of this study was to examine changes in body composition, energy metabolites and electrolytes during a 10-day winter survival training period. Two groups of male soldiers were examined: the REC group (*n* = 26; age 19.7 ± 1.2 years; BMI 23.9 ± 2.7) had recovery period between days 6 and 8 in the survival training, whereas the EXC group (*n* = 42; age 19.6 ± 0.8 years; BMI 23.1 ± 2.8) did not. The following data were collected: body composition (bioimpedance), energy balance (food diaries, heart rate variability measurements), and biomarkers (blood samples). In survival training, estimated energy balance was highly negative: −4,323 ± 1,515 kcal/d (EXC) and −4,635 ± 1,742 kcal/d (REC). Between days 1 and 10, body mass decreased by 3.9% (EXC) and 3.0% (REC). On day 6, free fatty acid and urea levels increased, whereas leptin, glucose and potassium decreased in all. Recovery period temporarily reversed some of the changes (body mass, leptin, free fatty acids, and urea) toward baseline levels. Survival training caused a severe energy deficit and reductions in body mass. The early stage of military survival training seems to alter energy, hormonal and fluid metabolism, but these effects disappear after an active recovery period.

## Introduction

In military survival training, soldiers are exposed to multiple physiological, psychological and environmental stressors for several days. In a physiological point of view, military survival training is associated with high-energy expenditure, restricted energy intake and limited sleep, and can cause remarkable changes in body composition, energy metabolism, hydration status and endocrinological stress function. Negative energy balance has been shown to lead to decreases in body mass, fat mass and fat free mass (Hoyt et al., 2006; Nindl et al., 2007; Hamarsland et al., 2018). Furthermore, many studies have observed a decline in physical performance during survival training (Nindl et al., 2007; Margolis et al., 2014; Hamarsland et al., 2018; Różański et al., 2020).

Military survival training has also been found to disturb hormonal regulation (Lieberman et al., 2016; Szivak et al., 2018). Leptin is an adipose-derived hormone, which regulates appetite and energy metabolism (Pasiakos et al., 2011). Leptin concentration decreases during starvation (Ahima et al., 1996; Chan et al., 2003) and in military training that involves severe energy restriction (Pasiakos et al., 2011). During a 6-month crisis management operation, leptin concentration has been reported to correlate positively with body fat% (Hill et al., 2015). Another appetite-regulating hormone, ghrelin, is secreted from the gut and duodenum, and stimulates appetite (Pasiakos et al., 2011). *In vivo* ghrelin circulates in acylated and unacylated forms, and the latter form accounts for over 90% of total circulating ghrelin (Kojima et al., 1999). During an acute energy deficit, ghrelin concentration typically increases, but is not related to perceived satiety (Pasiakos et al., 2011).

When energy deficit lasts for several days, lipolysis and protein degradation may occur to maintain substrate metabolism at an appropriate level (Cahill, 2006). Glucose is an essential substrate for the brain, but when carbohydrate intake is insufficient, gluconeogenesis is upregulated to ensure adequate blood glucose level and glucose availability for the cells (Cahill, 2006). Degradation of adipose tissue provides energy for skeletal muscles, especially when glycogen stores are empty and carbohydrates are not available. Thus, loss of skeletal muscle is common during military training due to energy deprivation (Hoyt et al., 2006; Nindl et al., 2007; Hamarsland et al., 2018). In addition, loss of whole-body protein has been observed (Margolis et al., 2014). Serum creatinine is a biomarker that typically indicates renal function, but as a product of muscle catabolism, it has also been used to estimate the volume of muscle mass (Kim et al., 2016; Delanaye et al., 2017).

A rapid decrease in body mass can be explained by dehydration and/or fluid loss. In military survival training, hydration status may change rapidly, whereby restricted fluid and food intake, combined with continuous exercise, cause fluid loss. Blood biomarkers, such as sodium, have been used to evaluate hydration status (Nolte et al., 2019). In prolonged exercise, hypernatremia may occur due to a loss of total body water. A more severe sodium imbalance, hyponatremia, often results from decreased sodium and potassium, a relative excess of total body water, or a combination of both.

Metabolic changes that occur during strenuous prolonged exercise can also be estimated *via* blood parameters (Warburton et al., 2002). Only a few studies have explored these metabolic responses in a military training context (Hill et al., 2015; Karl et al., 2017). Furthermore, physiological responses of recovery period during survival trainings are poorly understood. Thus, the purpose of the present study was (a) to examine the effect of a 10-day winter survival training period on body composition, energy balance, appetite-mediating hormones, substrate metabolites and electrolytes; and (b) to compare changes in biomarkers between an exercise (EXC) and a recovery (REC) group.

## Materials and Methods

Sixty-eight male soldiers participated in the study, and they were divided into two groups: REC (*n* = 26) and EXC (*n* = 42). The division was done according to the platoons of the participants, which facilitated the planning of education. More soldiers were directed to the EXC group because a higher drop-out rate was anticipated. In the REC group 20 participants passed through the training and in the EXC group 25. The most common reasons for drop-outs were musculoskeletal disorders and upper respiratory tract infections. Three female soldiers were excluded in the EXC group, since there were no women in the REC group. Basic characteristics of the participants are presented in Table 1.

**TABLE 1:** Basic characteristics of the participants.

Characteristics	REC (*n* = 26)	EXC (*n* = 42)
Age (years)	19.7 ± 1.2	19.6 ± 0.8
Height (cm)	181.1 ± 5.8	179.4 ± 6.2
Body mass (kg)	78.2 ± 9.6	74.4 ± 10.7
BMI (kg/m^2^)	23.9 ± 2.7	23.1 ± 2.8

*Values are presented as means ± SD. No statistical differences between groups were found at baseline. REC, recovery group; EXC, exercise group, BMI, body mass index.*

The present study was approved by the Finnish Defence Forces (AO1720) and the ethical approval was granted by the Scientific and Ethical Committee of the Helsinki University Hospital Research (HUS/900/2018). All participants were informed of the experimental design, the methods, the benefits and possible risks prior to signing an informed consent document to voluntarily participate in the study. The study was a part of a larger multidisciplinary research project.

### Study Protocol

**FIGURE 1:**
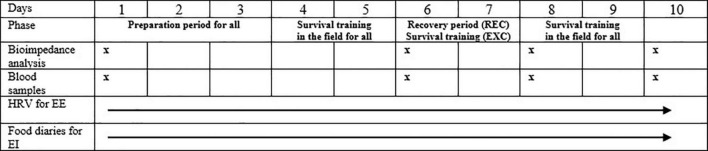
Study protocol. The REC group had 2 days recovery period, whereas the EXC group was in the field all the training. HRV, heart rate variability; EE, estimated energy expenditure, EI, energy intake.

The 10-d winter survival training, which included a 3-day preparation period, 6 days of survival training for the EXC group and a 1-day follow-up (for the REC group day 6 and 7 of the training was replaced with recovery) was carried out in March-April north of the Arctic Circle. The study protocol and measurements are presented in Figure 1. The first measurements (baseline, day 1) were conducted from 05:30 am (body composition, blood samples). Recordings of heart rate variability (HRV) and food diaries were also started later in the same morning. After the baseline measurements, a three-day preparation period began in the garrison. The participants slept in the garrison, and ate their breakfast, lunch, dinner and evening meal in a canteen. On day 4, all participants started their field training period. They performed different military tasks, slept in temporary shelters and carried their personal equipment in backpacks and pulks (extra load 23–32 kg). Participants moved with military cross-country skis in the field. Some of the military tasks were performed in subgroups including land navigation. Deep and soft snow made skiing harder than normal, and it was impossible to move without skis. The distance covered by skiing was on average 19.3 ± 1.7 km/day (min. 3.9 km; max. 25.8 km) during the field training period for the whole group. Daily variation of skiing distances occurred due to military tasks and a performance of land navigation. Education during the field training was identical for both groups, although some of military tasks were performed in smaller sub-groups. On day 6, participants were moved back to the garrison for the measurements, after which the REC group had a 2-day supervised recovery period while the EXC group returned to field training. On day 8, measurements were repeated before all participants returned to the field for the last 2 days, where circumstances were as previously described. Participants returned to the garrison late on day 9. In the morning of the day 10, post measurements were performed. All the measurements were carried out at the same protocol and the same timing for each measurement point.

### Field Conditions

In this survival training, energy intake was restricted on purpose. Modified field rations were delivered to the participants for the first 2 days of the field phase, consisting of a protein bar (226 kcal), eight crackers (306 kcal) and two lunch meal rations. Mean energy content of meal rations was 661 kcal, and one portion consisted of approximately 88 g carbohydrates, 27 g protein and 21 g fat. Total energy content of these items was 1,847 kcal. Drinking was allowed *ad libitum*, but the drinking water had to melt from snow. One educational theme was how to get food from nature, thus extra meals were prepared from reindeer meat, salmon and beard moss lichen (*Bryoria fuscescens*). Beard moss lichen was collected from the surface of spruce trees, then dissolved in water with sodium bicarbonate (baking soda) for a few hours and boiled for an hour. After 2 days, extra rations were delivered to the EXC group, whereas the REC group was evacuated for 2 days and given temporary accommodation in the training area. During the recovery period, they were given normal meals and some extra snacks (candies, beverages, cookies, ice cream). They had the possibility to go to the sauna and shower, and sleeping facilities were like those in a garrison. During the recovery period the participants had light supervised physical activity (ball games, stretching) and various psychological therapy sessions.

Self-reported sleeping times varied between 0.5 and 4 h per day in the field (Vaara et al., 2020). In the garrison and during the recovery period, 7–8 h of sleep per day was possible.

The weather was typical for late winter in Northern Finland, as temperatures increased by several degrees from night (min −10.5°C) to day (max 5.4°C). The mean temperature varied between −0.3°C and −4.7°C. The average depth of snow was between 80 and 100 cm. Overall description of the study protocol and field conditions has been published previously (Vaara et al., 2020).

### Blood Samples

Blood samples for leptin, unacylated ghrelin, creatinine, glucose, urea, free fatty acids and electrolytes (Na^2+^, K^+^, Cl^–^) were drawn from the antecubital vein after overnight fasting. Samples were collected into VenoSafe plastic tubes (VenoSafe^®^, Terumo Europe, Leuven, Belgium) containing silica gel. Blood samples were centrifuged (3,500 rpm, 10 min) and serum was frozen at −20°C for later analysis. Leptin and ghrelin were determined with an ELISA-kit immunoassay system (Dynex DS 2, Dynex Technologies, Chantilly, VA, United States); creatinine, glucose and urea with a photometric enzymatic method (Konelab 20 Xti Clinical Chemistry Analyzer, Thermo Scientific, Vantaa, Finland); free fatty acids with an enzymatic colorimetric assay (Konelab 20 Xti Clinical Chemistry Analyzer, Thermo Scientific, Vantaa, Finland); and electrolytes (Na, K, Cl) with an ion selective electrode method (ISE; Konelab 20 Xti Clinical Chemistry Analyzer, Thermo Scientific, Vantaa, Finland).

The sensitivity and inter-assay coefficient of variation for these assays were: 0.2 ng/ml, 6.1% for leptin; 0.6 pg/ml, 17.3% for ghrelin; 2.32 μmol/l, 2.2% for creatinine; 0.1 mmol/l; 3.8% for glucose; 1.1 mmol/l, 5.8% for urea, 10 μmol/l, 5.8% for free fatty acids; 100 mmol/l, 0.7% for sodium; 2 mmol/l, 2.5% for potassium and 55 mmol/l, 3.3% for chloride.

### Body Composition Assessment

Body composition variables (body mass, body fat%, skeletal muscle mass) were evaluated *via* bioimpedance devices (Inbody 720/770, Biospace, Soul, South Korea). The measurements were done early in the morning after an overnight fast, and participants were advised to only wear underwear and to urinate before the measurement. The same device was used for each measurement to avoid variability between devices.

### Estimated Energy Expenditure and Intake

Energy expenditure was estimated *via* heart rate variability measurements (Firstbeat Bodyguard 2, Firstbeat Technologies Oy, Jyväskylä, Finland). The Bodyguard 2 is a two-electrode portable device connected to the chest. Participants wore the Bodyguard 2 continuously, with the exception of short breaks to install the batteries. The accuracy of estimated energy expenditure is within 7–10% (Smolander et al., 2011). Pre-filled food diaries were used to estimate energy intake. Since food intake was restricted during the field training on purpose and delivered food items were known beforehand, participants were given pre-filled food diaries, where they wrote the time and amount of food consumed, including also extra food delivered in the field. Nevertheless, some of the diaries were too inaccurate, were lost or got wet in the field, so only representative diaries from days 5 and 7 were included for further analysis. Energy intake was calculated according to the nutrition value of each food item and energy content of extra food, which were analyzed by software program for obtaining individual total energy intake (Fineli, National Food Composition Database, Finland).

### Statistical Analysis

Time × group interactions were tested with F-tests based on the Satterthwaite method using the lmerTest R-package (Kuznetsova et al., 2017). A linear mixed effect model was used to estimate changes within and between groups over the studied period. Since military survival training is strenuous, failure and drop-out rates are relatively high. Therefore, a linear mixed effect model was chosen instead of repeated measures ANOVA to maximize observations at each time point. Pairwise comparisons were performed using Tukey’s test and logarithmic transformations were done when the distribution was positively skewed (leptin, ghrelin, free fatty acids). All data were examined quantitatively and graphically. Non-parametric Mann–Whitney *U*-tests were used to verify the linear mixed effect model when residuals were not normally distributed (body mass, leptin, glucose, sodium and chloride). Spearman correlations were calculated to estimate associations at baseline and differences in associations from baseline to day 10. All statistical analyses were performed using R v. 3.6.3 (2020, R Foundation for Statistical Computing, Vienna, Austria). Data are presented as means ± standard deviations and statistical significance was set at *p* < 0.05.

## Results

Estimated energy expenditure was high in both groups during the training. In Table 2, energy expenditure values from day 2 to day 9 are presented for both groups. On days 1 and 10, an entire 24-h recording was not possible, so these values were excluded.

**TABLE 2:** Mean ± SD daily values of energy expenditure (kcal/d) in the exercise (EXC) and recovery (REC) groups.

	Day 2	Day 3	Day 4	Day 5	Day 6	Day 7	Day 8	Day 9
EXC	3,369 ± 670 *n* = 40	5,469 ± 1,593 *n* = 40	4,389 ± 2,008 *n* = 28	4,407 ± 1,850 *n* = 31	5,717 ± 1,162 *n* = 26	5,521 ± 1,204 *n* = 27	5,113 ± 1,592 *n* = 28	3,570 ± 1,202 *n* = 25
REC	3,690 ± 646 *n* = 25	5,546 ± 1,318 *n* = 25	4,340 ± 1,936 *n* = 23	4,978 ± 1,540 *n* = 22	4,998 ± 1,406 *n* = 22	3,517 ± 887 *n* = 22 ^‡‡‡^	5,621 ± 1,192 *n* = 23	4,162 ± 1,005 *n* = 21

*^‡‡‡^p < 0.001, between groups.*

Based on the representative food diaries from both groups, energy intake was 447 ± 245 kcal/d (EXC, *n* = 24) and 357 ± 338 kcal/d (REC, *n* = 20) on day 5, and 1,294 ± 743 kcal/d (EXC, *n* = 12) and 3,003 ± 882 kcal/d (REC, *n* = 21) on day 7. Energy intake differed statistically significantly between groups (*p* < 0.001) on day 7. Calculated energy balance was −4,323 ± 1,515 kcal/d in the EXC group and −4,635 ± 1,742 kcal/d in the REC group on day 5. On day 7, the estimated energy balance was −4,222 ± 1,815 kcal/d (EXC) and −608 ± 1,107 kcal/d (REC), and the difference was statistically significant (*p* < 0.001).

For body composition parameters, significant time × group interactions were found for body mass (*p* < 0.001), body fat% (*p* < 0.001) and skeletal muscle mass (*p* = 0.004) but not for creatinine. Differences in model parameters within and between groups are presented in Table 3. Body mass decreased in the EXC group by day 6 and remained lower than baseline thereafter. In the REC group, body mass first decreased by day 6 but then increased by day 8, and finally decreased again by day 10. On day 8, significant differences were found in body mass (*p* < 0.001) and body fat% (*p* = 0.017) between the groups, and on day 10, body fat% also differed (*p* < 0.001) between the groups.

**TABLE 3:** Changes in body mass, body fat%, skeletal muscle mass and serum creatinine during the training period.

		Day 1	Day 6	Day 8	Day 10	Δ (%) day 1–10
Body mass (kg)	EXC	74.4 ± 10.7	72.9 ± 9.8 *p* < 0.001 ^day1_6^	72.6 ± 9.6 *p* < 0.001 ^day1_8^	72.6 ± 9.5 *p* < 0.001 ^day1_10^	−3.9 ± 1.7
	REC	78.2 ± 9.7	74.6 ± 9.2 *p* < 0.001 ^day1_6^	77.1 ± 8.6 *p* < 0.001 ^day1_8^ *p* < 0.001 ^day6_8^	75.5 ± 9.0 *p* < 0.001 ^day1_10^ *p* < 0.001 ^day6_10^ *p* < 0.001 ^day8_10^	−3.0 ± 2.1
Body fat%	EXC	13.8 ± 4.8	12.8 ± 3.9 *p* < 0.001 ^day1_6^	10.4 ± 3.3 *p* < 0.001 ^day1_6^ *p* < 0.001 ^day6_8^	9.0 ± 3.5 *p* < 0.001 ^day1_10^ *p* < 0.001 ^day6_10^ *p* < 0.001 ^day8_10^	−37.0 ± 9.6
	REC	14.1 ± 5.2	11.5 ± 5.6 *p* < 0.001 ^day1_6^	10.9 ± 4.7 *p* < 0.001 ^day1_8^	10.9 ± 4.7 *p* < 0.001 ^day1_10^	−20.1 ± 11.4
Skeletal muscle mass (kg)	EXC	36.2 ± 4.6	35.9 ± 4.8 *p* < 0.001 ^day1_6^	36.9 ± 4.8 *p* < 0.001 ^day6_8^	37.3 ± 4.8 *p* < 0.001 ^day6_10^	1.4 ± 2.5
	REC	38.0 ± 3.7	37.1 ± 3.1 *p* < 0.001 ^day1_6^	38.6 ± 3.2 *p* < 0.001 ^day6_8^	38.0 ± 3.3 *p* < 0.001 ^day6_10^ *p* = 0.003 ^day8_10^	−0.2 ± 2.5
Creatinine (μmol/l)	EXC	89.8 ± 11.4	82.4 ± 12.2 *p* < 0.001 ^day1_6^	88.4 ± 11.9 *p* = 0.017 ^day6_8^	91.9 ± 10.1 *p* < 0.001 ^day6_10^	2.2 ± 10.2
	REC	89.2 ± 9.4	85.2 ± 12.6	91.1 ± 8.4	91.6 ± 9.2 *p* = 0.031 ^day6_10^	2.1 ± 12.5

*For body mass, body fat% and skeletal muscle mass n = 42, 38, 27, 25 (EXC) and n = 26, 22, 22, 20 (REC) for days 1, 6, 8 and 10 respectively. For creatinine n = 32, 26, 27, 25 (EXC, respectively) and n = 20, 18, 18, 18 (REC). Significant differences are presented within the groups.*

**FIGURE 2:**
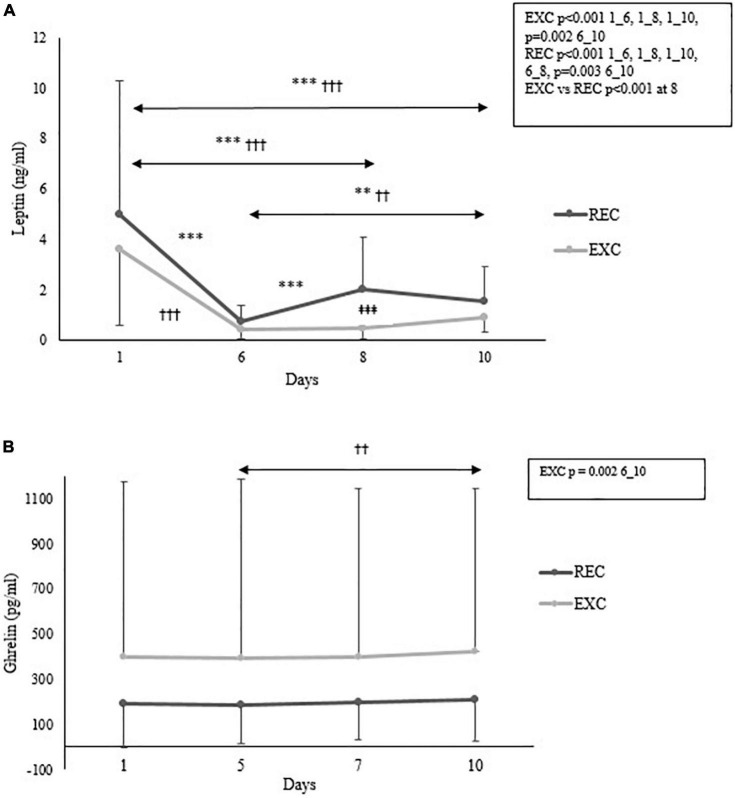
Changes in serum **(A)** leptin and **(B)** ghrelin concentrations within and between the REC and EXC groups. For leptin, a number of subjects in the REC and EXC groups were daily 26, 22, 22, 20 and 42, 26, 27, 25, respectively. For ghrelin *n* = 17 (on all days) in the REC group and *n* = 23, 23, 23, 22 in the EXC group for days 1, 6, 8, 10 respectively. *REC, ^†^EXC, and ^‡^Between groups. ^**^,^††^*p* < 0.01; ^†††^,^‡‡‡^,^***^p < 0.001.

The time × group interaction for leptin was statistically significant (*p* < 0.001), but not for ghrelin. Serum leptin concentration decreased significantly in both groups from day 1 to day 6, while in the EXC group, it stayed at the lower level until the end of the study (Figure 2A). After the recovery period, leptin concentration increased in the REC group toward baseline so that a significant difference between the groups was observed on day 8. Serum ghrelin level stayed stable throughout the studied period, except for a slight increase in the EXC group from day 6 to day 10 (Figure 2B).

**FIGURE 3:**
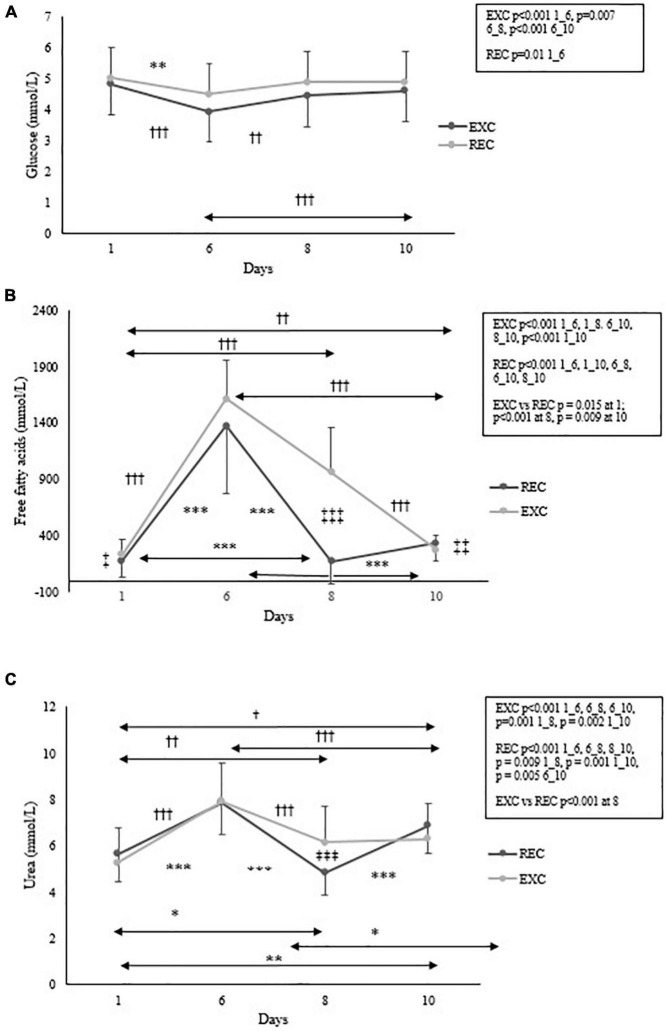
Changes in **(A)** glucose, **(B)** free fatty acids and **(C)** urea concentrations within and between the REC and EXC groups. In the REC group, *n* = 26, 22, 22, 20 and in the EXC group *n* = 42, 26, 27, 25 for days 1, 6, 8, 10 respectively. *REC, ^†^EXC, and ^‡^Between groups. *,^†^,^‡^*p* < 0.05; ^**^,^††^,^‡‡^*p* < 0.01; ^***^,^†††^,^‡‡‡^*p* < 0.001.

For energy substrate metabolites, significant time × group interactions were found for free fatty acids (*p* < 0.001) and urea (*p* < 0.001) but not for glucose. Results for these biomarkers are presented in Figure 3. Significant decreases in glucose were found at day 6 in both groups, followed by a slight increase only in the EXC group toward the end of the study. Relative to baseline, free fatty acid concentration increased at day 6 by 670% (REC) and 597% (EXC), but the recovery period resulted in the return of free fatty acid concentration toward baseline in the REC group (no difference between days 1 and 8 and a significant difference between the groups). Several significant changes were observed in urea concentration within the groups at all phases of the training, but the only significant difference between the groups was found at day 8.

**FIGURE 4:**
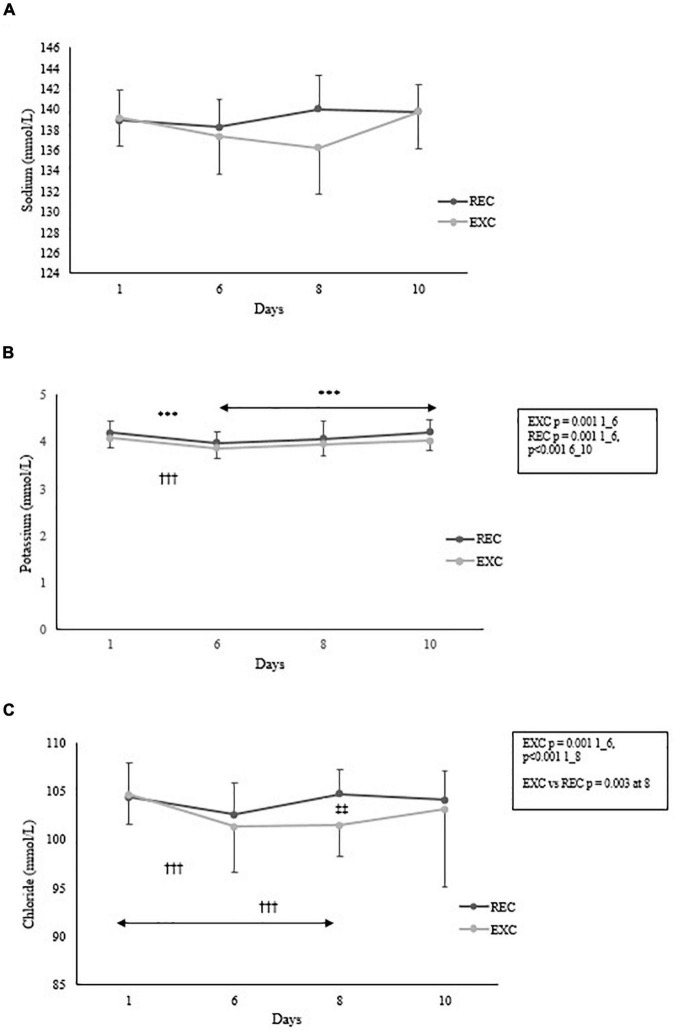
Changes in **(A)** sodium **(B)** potassium and **(C)** chloride concentration within and between the REC and EXC groups. In the REC group *n* = 26, 22, 22, 20 and in the EXC group *n* = 42, 26, 27, 25 for days 1, 6, 8, 10 respectively. *REC, ^†^EXC, and ^‡^Between groups. ^‡‡^*p* < 0.01; ^***^,^†††^*p* < 0.001.

For serum electrolytes, significant time × group interactions were found for sodium (*p* = 0.002) and chloride (*p* = 0.03) but not potassium. Concentrations of these electrolytes (Na, K, and Cl) are shown in Figure 4. No significant changes in sodium concentration were found, whereas in the EXC group chloride concentration was significantly lower than baseline at days 6 and 8, and the groups differed significantly at day 8. Potassium levels decreased in both groups by day 6, and then slightly increased, but the increase was only significant in the REC group.

At baseline, strong positive associations were found between body mass and skeletal muscle mass (*r* = 0.911, *p* < 0.001), body mass and leptin (*r* = 0.573, *p* < 0.001), body mass and body fat% (*r* = 0.500, *p* < 0.001), and leptin and body fat% (*r* = 0.737, *p* < 0.001). When evaluating correlations between differences from day 1 to day 10, a strong inverse association was observed between skeletal muscle mass and body fat% (*r* = −0.682, *p* < 0.001). No other systematic associations were found within biomarkers or between changes in different biomarkers and body composition variables. Correlation matrices at baseline and in differences from day 1 to 10 for the whole group are presented in Supplementary Digital Content 1.

## Discussion

The main findings of this study were (a) winter survival training caused severe energy deficit, which contributed to decreases in body mass and body fat%, serum leptin, glucose and potassium concentration, and increases in free fatty acids and urea; (b) a 2-day recovery period during survival training temporarily reversed some of the changes (body mass, leptin, free fatty acids, urea) toward baseline levels, but body mass, body fat% and leptin did not fully recover.

Estimated energy balance was highly negative in both groups during the training and recovery periods, as also evidenced by a decrease in body mass. Participants in the REC group were able to eat and drink *ad libitum* during the 2-day recovery period but still their energy balance stayed negative. It should be noted that energy expenditure was estimated *via* continuous monitoring of heart rate variability and energy intake *via* self-reported food diaries, which both may feature inaccuracies (Capling et al., 2017; Fuller et al., 2020). The REC group had still high energy expenditure values during their recovery period, partly explained by the stress of cardiovascular and autonomic nervous system. Compared to previous literature, Kyröläinen et al. (2017) reported an energy deficit of ∼4,000 kcal during the first week of military field exercise. In Norwegian soldiers, the energy deficit was approximately 2,900 kcal/d during winter military training (Margolis et al., 2014), and in Naval Special Warfare SEAL Qualification Students the deficit was 1,044–3,112 kcal/d (Beals et al., 2019). Survival training in the present study differed from previous studies whereby food intake was deliberately restricted, causing remarkable physiological and psychological stress to participants. Without dietary manipulation, military training often produces a negative balance, which needs to be acknowledged and minimized, if possible. According to Beals et al. (2019), focusing on macronutrient supply and providing extra snacks for recovery periods will compensate for the lack of energy in intense military training. Even in harsh circumstances (Greenland expedition), energy balance can be maintained if the food supply is appropriate and well planned (Gagnon et al., 2011; Charlot et al., 2020).

As hypothesized, body mass and body fat% decreased during the training period. In the EXC group, body fat% declined throughout the 10-day period but in the REC group body fat% remained stable between days 6 and 10. Loss of body mass is typical in survival training. For example, Hoyt et al. (2006) reported a loss of 7.7 ± 1.1 kg and a decrease in fat free mass in male cadets during a 7 day ranger field exercise. Hamarsland et al. (2018) noted a 5.3 ± 1.9 kg decrease in muscle mass during Norwegian Special Forces’ “hell week,” after which body mass returned to baseline within 1 week. Magnitude of weight loss is associated with the intensity and duration of exercise, the magnitude of energy and fluid deprivation, and the length of field training. In all the above-mentioned studies, body mass at baseline was approximately the same (78 kg) and BMI was in the normal range, making higher losses of body mass more critical for lean soldiers.

Interestingly, skeletal muscle mass (estimated by bioimpedance) decreased by day 6 but then increased toward the end of the study in both groups. Previous findings indicate that fat free mass typically decreases in severe energy deficit (Hoyt et al., 2006; Nindl et al., 2007; Hamarsland et al., 2018). Bioimpedance measurements were performed in the mornings after an overnight fast, but we suspect that the dehydrated state of participants may have interfered with the results. Therefore, we also measured serum creatinine to clarify the body composition results. Creatinine is a biomarker of renal function, but it can also be used as a proxy for the amount of muscle mass (Kim et al., 2016; Delanaye et al., 2017). In this study, creatinine reacted in the same way as skeletal muscle mass, whereby its concentration increased from day 6 to day 10. The REC group did consume a higher amount of energy during their recovery period, but the EXC group also got additional energy from day 6 onward. A remarkable energy deficit still existed. One explanation for these inverse muscle mass results could be a protective metabolic mechanism during prolonged energy deprivation, where cells uptake all of the amino acids available for protein synthesis, and lipids become the primary energy substrate (Gagnon et al., 2020). Ocobock (Ocobock, 2017) suggested that the amount of body fat may protect muscle mass during a state of negative energy balance, since females gained muscle mass but males lost it in their study. Another explanation for the results is that, the used methodologies were interfered by dehydrated and unfed state of participants and factually muscle mass did not increase.

Severe energy deficit, loss of body mass and especially loss of fat free mass have detrimental consequences for soldiers, since they disturb muscle function, the endocrinological system and military performance (Pasiakos, 2020). During arduous training, macronutrient supplementation (protein, carbohydrates) has been used to help maintain fat free mass and to provide extra energy (Pasiakos, 2020). Some novel methods have also been examined, for example, the use of ketones for extra fuel (Margolis and O’Fallon, 2020) and low-dose testosterone supplementation for preventing hormonal disturbances (Pasiakos et al., 2019), but more studies are needed to clarify the safety, dose, timing and side effects of these supplements.

A decline in leptin concentration is typically associated with acute and severe energy deficit (Pasiakos et al., 2011). In the present study, a clear decrease in serum leptin was observed at day 6 in both groups. At baseline, leptin was positively associated with body mass and body fat%, as observed in previous studies (Pasiakos et al., 2011; Hill et al., 2015). Although leptin concentration is associated with adipose tissue, the regulation of leptin concentration may alter *via* glucose metabolism (Pasiakos et al., 2011). *In vitro* and *in vivo* studies have demonstrated that blood glucose regulates leptin gene expression, but short-term starvation does not necessarily mediate leptin gene expression; it merely decreases circulating leptin concentration (Kolaczynski et al., 1996; Pasiakos et al., 2011). In this study, fasting blood glucose levels were reduced at day 6 in both groups. The training led to low glucose concentrations for several days, which could diminish leptin gene expression and circulating leptin concentration, although no associations were found.

Ghrelin is an appetite-regulating hormone and in the current study, serum ghrelin concentration remained stable throughout the training, except for a small increase in the EXC group from day 6 to day 10. These findings agree with those of a previous study, where ghrelin concentration was assessed during short-lasting starvation (Pasiakos et al., 2011): ghrelin concentration was elevated and stable during energy deficit, whereas it decreased acutely in response to feeding. In the present study, although the REC group consumed food and drinks *ad libitum* during their recovery period, this did not affect ghrelin concentration, likely because of inappropriate timing of blood samples. Hill et al. (2015) reported that ghrelin levels were elevated in soldiers who lost the greatest amount of body mass during military deployment, but this phenomenon was not seen in the current study. A few particularly high values of ghrelin were measured in the EXC group, but they were included in the statistical analysis, since elevated values were found from the same participants at all four measurement points systematically.

Blood urea concentration indicates the breakdown of nitrogen containing compounds, which typically means degradation of protein (Haralambie and Berg, 1976). During the first 2 days in the field, when energy intake was severely restricted, glycogen stores were likely depleted and muscle protein degraded. Urea concentration slightly increased, as well as free fatty acids, whereas blood glucose concentration decreased at day 6 in both groups. Free fatty acid levels peaked at day 6 in both groups and these increases were as high as 670% (REC) and 597% (EXC). According to Cahill (2006), starvation modifies fuel metabolism to ensure continuous glucose transportation to the brain. In a post-absorptive state, blood glucose and glycogen are utilized and if starvation lasts for several days, gluconeogenesis is activated. At the same time, free fatty acids are released into the bloodstream, which indicates lipolysis in adipose tissue. In the current study, the most dramatic change in energy metabolism occurred after 2 days of field training, where only a small amount of energy (357–447 kcal/d) was consumed. The lack of dietary carbohydrate shifted the primary fuel source from glycogen to fat. When additional energy was given, the biomarkers of energy substrates recovered toward baseline and muscle mass slightly increased, implying that muscle protein was spared and that all dietary protein was utilized for protein synthesis. In a study by Karl et al. (2017), a large variety of metabolites were measured during a military cross-country ski march that induced a severe energy deficit. Clear increases in fat metabolism were observed, as well as moderate increases in tricarboxylic acid cycle intermediates and branched chain amino acid metabolites. The magnitudes of changes in protein metabolites were not as high as for lipid metabolites. In the present study, only a few biomarkers of energy metabolism were used, but the findings agree with the previously described fuel metabolism results during starvation.

Rapid weight loss is usually explained by fluid loss. Sodium is the major cation in the intracellular space (Warburton et al., 2002). Hyponatremia is diagnosed when blood sodium concentration is below 135 mmol/l, and a concentration below 130 mml/l indicates severe hyponatremia. In the EXC group, sodium concentration was almost hyponatraemic at day 8 (136 mmol/l). Statistically significant changes were not observed within or between groups, mainly due to individual variation and high SD of the samples. In ultra-endurance events, hyponatremia is the principal electrolyte disorder, and commonly found in athletes seeking medical help after prolonged strenuous exercise. Loss of sweat and overhydration may be the main factors responsible for hyponatraemia. The present participants were likely dehydrated due to high sweat loss and restricted fluid intake, and this is supported by the observed rapid loss of body mass. Dehydration lowers the extracellular fluid volume, which may compensate for the sodium concentration in the blood (Pedlar et al., 2019).

Potassium concentration decreased during the first 6 days of training in both groups. Potassium is the major electrolyte in intracellular fluid, and it is released to the extracellular space during exercise in direct proportion to exercise intensity (Warburton et al., 2002). Hyperkalaemia is an acute response to exercise, but reuptake of potassium into the muscle may further lead to a hypokalaemic state. In the current study, an acute effect was not observed, and the training was longer than a typical ultra-endurance event. Nonetheless, we assume that part of the participants was heading to hypokalemia after the most strenuous phase of training at day 6.

In addition to sodium, chloride is another ion that can be lost *via* sweating in the form of sodium chloride (Emenike et al., 2014). At the cellular level, sodium and chloride ions usually move in the same direction from extracellular fluid into the cells. Thus, sodium and chloride concentrations tend to be correlated, but this was not the case in this study. Chloride concentration was lower in the EXC group at day 8, which may indicate a loss of electrolytes due to prolonged exercise. However, Gagnon et al. (2020) did not find any significant changes in electrolytes (Na^2+^, K^+^, Cl^–^) after a 14-day canoeing expedition (compared to the control group), although they did not compare to baseline values and the study protocol was not comparable.

Based on the findings of this study, soldiers were exposed different physiological stress factors during survival training, which disturbed body’s homeostasis, especially during the first days of strenuous training. Short recovery period had a positive effect on part of the measured biomarkers, which can improve military performance of soldiers. To optimize performance in survival training, energy and fluid refueling, adequate sleep as well as mental preparation before the training, may enhance the body’s ability to adjust to arduous circumstances.

### Strengths and Limitations

Our study protocol had some limitations, which must be acknowledged. Evaluation of energy expenditure was based on continuous heart rate recordings. Although it was reported that the accuracy of this procedure is 7–10% (Smolander et al., 2011), validation was not done with double-labeled water or direct calorimetry. We did ask about fluid intake but the diaries were not sufficiently valid to report these results. This data could have helped in the interpretation of changes in body mass, muscle mass and electrolytes. Bioimpedance analysis is a simple method of assessing body composition outside of the laboratory environment, and it is known that it may overestimate fat free mass and underestimate fat mass in civilians (Sillanpää et al., 2014) and in a military context (Langer et al., 2016). Since the method is based on anthropometry and the fluid content of the body, rapid changes in hydration status may interfere with impedance values. However, changes in creatinine concentration occurred in parallel with bioimpedance results, which is exceptional in training that involves energy deprivation. A valid biomarker for protein metabolism was missing due to analytical problems with free amino acid concentrations. Thus, more accurate studies are needed to clarify protein and substrate metabolism during energy deficit. A high number of dropouts is typical for arduous training (Vaara et al., 2020), but by using a mixed statistical model, we were able to use the maximum number of observations. Despite the high dropout rate, an adequate number of participants finished the survival training in both groups. Acute and severe sleep deprivation may associate with part of these results, since glucose and appetite regulation (e.g., leptin, ghrelin) are influenced by sleep (Leproult and Van Cauter, 2010). Unfortunately, the importance of sleep deprivation has not been investigated in the present study, but further study is needed, especially in military population. It is still worth emphasizing the unique protocol and extraordinary caloric and sleep deprivation, which may not be possible among civilians.

### Conclusion

The present study showed that the most remarkable changes in metabolism occurred in the first 2 days of survival training, where the energy balance was highly negative. Changes in blood parameters partly recovered, especially when recovery was emphasized, but body mass and body fat% remained below baseline levels throughout the training period. A 2-day active recovery period in the middle of strenuous training may be sufficient to normalize body function and physical performance of soldiers.

## Data Availability Statement

The raw data supporting the conclusions of this article will be made available by the authors, without undue reservation.

## Ethics Statement

The studies involving human participants were reviewed and approved by the study was performed in line with the principles of the Declaration of Helsinki. The ethical approval was granted by the Scientific and Ethical Committee of the Helsinki University Hospital Research (HUS/900/2018). The patients/participants provided their written informed consent to participate in this study.

## Author Contributions

TN, TO, MF, and HK conceived and designed the research. TN and TO conducted experiments. TN, TO, and RH analyzed data. TN wrote the manuscript. All authors read and approved the manuscript.

## Conflict of Interest

The authors declare that the research was conducted in the absence of any commercial or financial relationships that could be construed as a potential conflict of interest.

## Publisher’s Note

All claims expressed in this article are solely those of the authors and do not necessarily represent those of their affiliated organizations, or those of the publisher, the editors and the reviewers. Any product that may be evaluated in this article, or claim that may be made by its manufacturer, is not guaranteed or endorsed by the publisher.
