# Supplementary material for: Changes in Body Composition, Energy Metabolites and Electrolytes During Winter Survival Training in Male Soldiers
Source: Front Physiol. 2022 Feb 16;13:797268. doi: 10.3389/fphys.2022.797268 (PMC8889070; doi:10.3389/fphys.2022.797268)
Supplement: Supplementary file 1 [file Table_1.docx]

Supplemental Digital Content 1.

Correlation matrix at baseline. Correlations (r) in upper triangle, statistical significances (p) in lower triangle.

FFA=free fatty acids, SMM=skeletal muscle mass

| **Variable** | **Chloride** | **FFA** | **Body fat %** | **Ghrelin** | **Glucose** | **K** | **Body mass** | **Creatinine** | **Leptin** | **Na** | **SMM** | **Urea** |
| --- | --- | --- | --- | --- | --- | --- | --- | --- | --- | --- | --- | --- |
| **Chloride** |  | -0.314 | -0.268 | -0.090 | 0.193 | 0.273 | -0.077 | 0.142 | 0.111 | 0.119 | 0.015 | 0.173 |
| **FFA** | 0.009 |  | 0.071 | -0.024 | -0.087 | -0.031 | 0.024 | 0.023 | -0.129 | 0.141 | 0.033 | 0.021 |
| **Body fat %** | 0.027 | 0.563 |  | -0.233 | -0.023 | -0.130 | 0.500 | 0.229 | 0.737 | 0.227 | 0.141 | 0.096 |
| **Ghrelin** | 0.579 | 0.881 | 0.147 |  | -0.131 | -0.053 | -0.008 | -0.074 | -0.269 | -0.151 | 0.021 | -0.004 |
| **Glucose** | 0.115 | 0.479 | 0.855 | 0.420 |  | 0.168 | 0.090 | 0.025 | 0.109 | -0.162 | 0.103 | -0.148 |
| **K** | 0.024 | 0.800 | 0.292 | 0.746 | 0.170 |  | 0.012 | -0.180 | -0.023 | 0.060 | 0.075 | 0.077 |
| **Body mass** | 0.531 | 0.846 | 0.000 | 0.960 | 0.468 | 0.920 |  | 0.333 | 0.573 | 0.346 | 0.911 | 0.294 |
| **Creatinine** | 0.317 | 0.869 | 0.103 | 0.651 | 0.860 | 0.200 | 0.016 |  | 0.302 | 0.040 | 0.295 | 0.269 |
| **Leptin** | 0.365 | 0.293 | 0.000 | 0.094 | 0.375 | 0.851 | 0.000 | 0.029 |  | 0.301 | 0.328 | 0.344 |
| **Sodium** | 0.333 | 0.250 | 0.062 | 0.353 | 0.188 | 0.629 | 0.004 | 0.778 | 0.013 |  | 0.292 | 0.340 |
| **SMM** | 0.905 | 0.790 | 0.252 | 0.899 | 0.405 | 0.544 | 0.000 | 0.034 | 0.006 | 0.016 |  | 0.321 |
| **Urea** | 0.159 | 0.866 | 0.435 | 0.979 | 0.230 | 0.530 | 0.015 | 0.053 | 0.004 | 0.005 | 0.008 |  |

Correlation matrix in differences from baseline to 10 d.

| **Variable** | **Chloride** | **FFA** | **Body fat %** | **Ghrelin** | **Glucose** | **K** | **Body mass** | **Creatinine** | **Leptin** | **Na** | **SMM** | **Urea** |
| --- | --- | --- | --- | --- | --- | --- | --- | --- | --- | --- | --- | --- |
| **Chloride** |  | 0.087 | -0.267 | 0.121 | 0.403 | 0.354 | -0.119 | -0.078 | -0.053 | 0.047 | 0.238 | 0.307 |
| **FFA** | 0.569 |  | 0.223 | 0.041 | 0.076 | 0.057 | -0.155 | 0.250 | -0.074 | 0.130 | -0.373 | 0.170 |
| **Body fat %** | 0.076 | 0.141 |  | 0.062 | -0.109 | -0.067 | 0.280 | 0.059 | 0.336 | 0.034 | -0.682 | 0.106 |
| **Ghrelin** | 0.461 | 0.806 | 0.706 |  | -0.061 | -0.032 | -0.255 | 0.110 | -0.160 | -0.095 | -0.236 | 0.283 |
| **Glucose** | 0.006 | 0.620 | 0.477 | 0.713 |  | 0.242 | -0.092 | -0.038 | 0.042 | -0.103 | 0.103 | 0.157 |
| **Potassium** | 0.017 | 0.708 | 0.661 | 0.846 | 0.109 |  | 0.368 | -0.250 | -0.008 | 0.029 | 0.332 | 0.250 |
| **Body mass** | 0.438 | 0.310 | 0.062 | 0.118 | 0.548 | 0.013 |  | -0.253 | 0.287 | 0.025 | 0.377 | 0.022 |
| **Creatinine** | 0.622 | 0.110 | 0.711 | 0.505 | 0.813 | 0.110 | 0.106 |  | 0.236 | 0.059 | -0.259 | 0.182 |
| **Leptin** | 0.727 | 0.631 | 0.024 | 0.331 | 0.783 | 0.956 | 0.056 | 0.132 |  | 0.061 | -0.164 | 0.144 |
| **Sodium** | 0.760 | 0.393 | 0.825 | 0.563 | 0.500 | 0.849 | 0.872 | 0.712 | 0.692 |  | -0.060 | -0.027 |
| **SMM** | 0.115 | 0.012 | 0.000 | 0.148 | 0.501 | 0.026 | 0.011 | 0.097 | 0.282 | 0.696 |  | -0.066 |
| **Urea** | 0.040 | 0.265 | 0.489 | 0.081 | 0.304 | 0.098 | 0.884 | 0.250 | 0.346 | 0.860 | 0.667 |  |
